# Supplementary material for: Structural role for DNA Ligase IV in promoting the fidelity of non-homologous end joining
Source: Nat Commun. 2024 Feb 10;15:1250. doi: 10.1038/s41467-024-45553-z (PMC10858965; doi:10.1038/s41467-024-45553-z)
Supplement: Supplementary file 1 — Supplementary Information [file 41467_2024_45553_MOESM1_ESM.pdf]

SUPPLEMENTARY INFORMATION

Structural role for DNA Ligase IV in promoting the fidelity of non-homologous end joining

Benjamin M. Stinson[1,2], Sean M. Carney[1], Johannes C. Walter[1,2,*], and Joseph J. Loparo[1,*]

[1]Department of Biological Chemistry and Molecular Pharmacology, Blavatnik Institute, Harvard Medical School, Boston, MA 02115, USA

[2]Howard Hughes Medical Institute, Boston, MA 02115, USA

*Correspondence:

johannes_walter@hms.harvard.edu (J.C.W)

joseph_loparo@hms.harvard.edu (J.J.L.)

Supplementary Data Figure 1

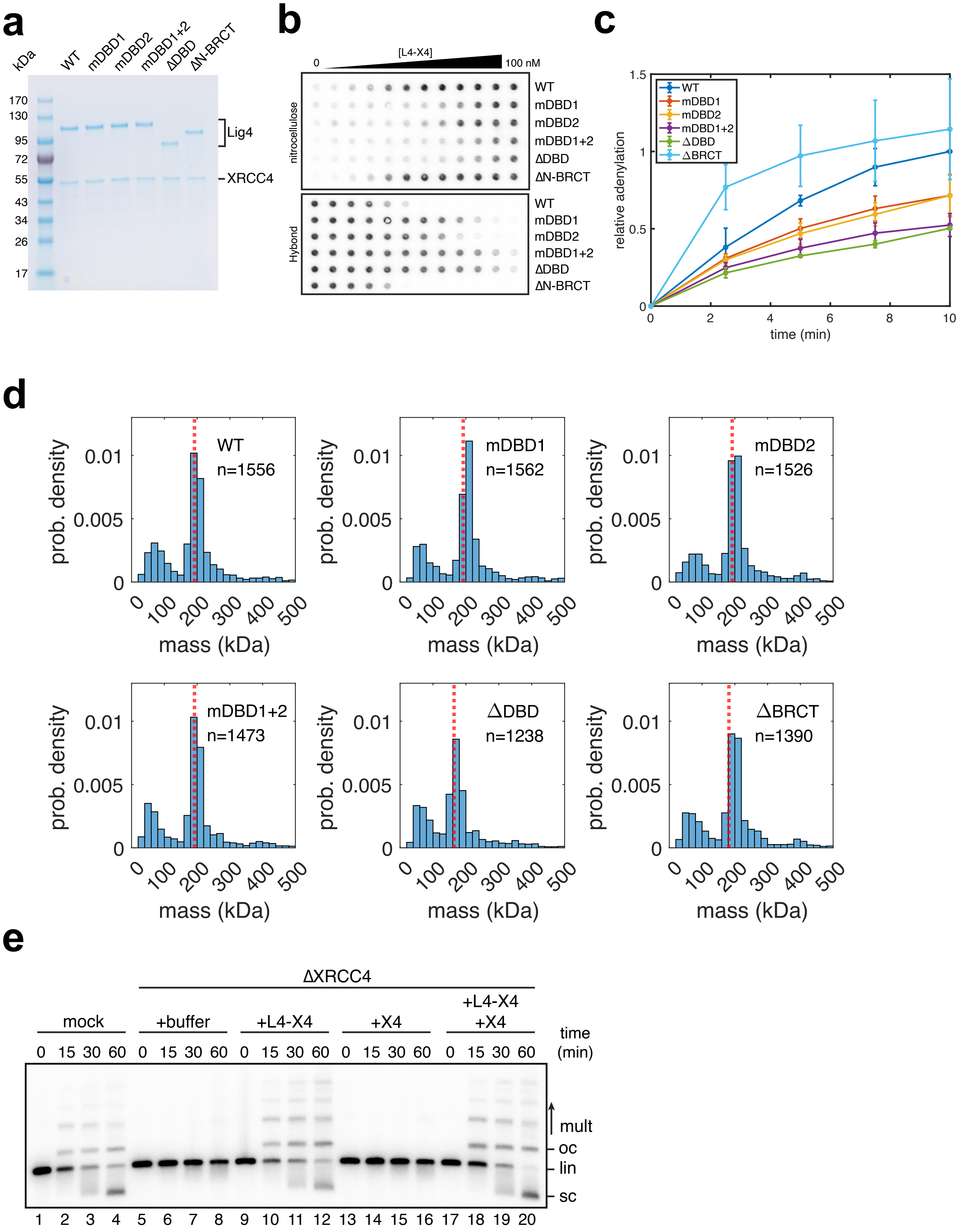

Supplementary Figure 1: Characterization of Lig4 DNA binding mutants, continued

(A) Coomassie-stained polyacrylamide gel of recombinantly purified Lig4-XRCC4 variants.

(B) Representative raw data for filter binding assay shown in Fig. 1b and described in Methods. Radiolabeled DNA bound by Lig4 is captured on the nitrocellulose membrane. Free DNA passes through the nitrocellulose membrane and is captured on the Hybond membrane. Lig4-XRCC4 concentration is a two-fold dilution series from 100 nM.

(C) Adenylation assay for Lig4 variants, as described in Methods. Cy5-labeled Lig4 variants were de-adenylated with inorganic pyrophosphate and re-adenylated using α-^{32}P-ATP and analyzed by SDS-PAGE and autoradiography. Extent of adenylation was normalized to the level of wild-type Lig4 adenylation at the 10 min timepoint. Error bars represent standard deviation from three independent experiments.

(D) Mass photometry assay to determine heterogeneity of recombinant Lig4-XRCC4 preparations. Dotted red line indicates expected mass of Lig4-XRCC4 complexes. The minor peak at ~60-80 kDa likely corresponds to un-complexed XRCC4 homodimer and/or Lig4-XRCC4 degradation products.

(E) Radiolabeled, blunt-ended, linear DNA molecules were added to the indicated extracts, and reaction samples were stopped at the indicated timepoints. Samples were analyzed by agarose gel electrophoresis and autoradiography. Where indicated, recombinant Lig4-XRCC4 was added to a final concentration of 50 nM, and un-complexed XRCC4 homodimer was added to a final concentration of 100 nM. lin: linear; sc: supercoiled; oc: open circular; mult: multimers. Three independent experiments were performed, and a representative autoradiogram is shown.

Supplementary Data Figure 2

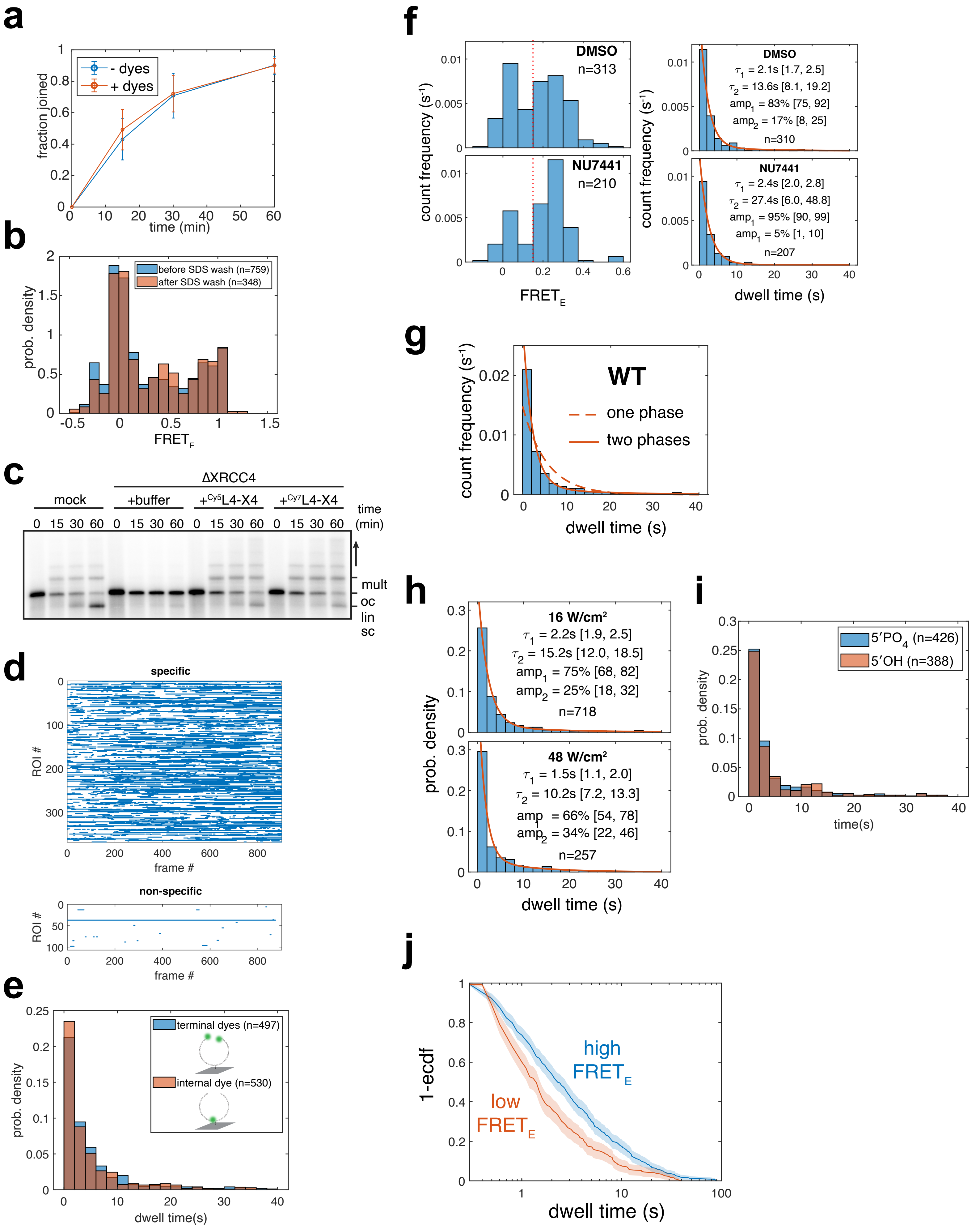

Supplementary Data Figure 2: DNA-binding by Lig4 is required for SR synapsis, continued.

(A) Radiolabeled, blunt-ended, linear DNA molecules either lacking fluorophores (-dyes) or with a Cy3B fluorophore 5 nt from one DNA end and a Cy5 fluorophore from the other end (+ dyes, as in Fig. 2a) were added to extracts, and reaction samples were stopped at the indicated timepoints. Samples were analyzed by agarose gel electrophoresis and autoradiography. Bands were quantified using ImageJ and fractional joining was calculated as 1-(linear monomer/total lane intensity). Data are represented as the mean of three independent experiments (error bars, standard deviation).

(B) FRET efficiency of immobilized Cy3B/Cy5-labeled DNA (as in Fig. 2a) before and after removal of protein complexes with a 1% SDS wash 5 min after introduction of extract.

(C) Radiolabeled, blunt-ended, linear DNA molecules were added to the indicated extracts, and reaction samples were stopped at the indicated timepoints. Samples were analyzed by agarose gel electrophoresis and autoradiography. lin: linear; sc: supercoiled; oc: open circular; mult: multimers. Three independent experiments were performed and a representative autoradiogram is shown.

(D) Rastergrams depicting specific and non-specific ^{Cy5}Lig4-XRCC4-^{Cy3B}DNA colocalization events from a representative experiment. Each row represents an individual DNA molecule, and blue lines represent times at which Lig4 colocalization was detected. Specific colocalizations occur at regions of interest (ROIs) containing ^{Cy3B}DNA signal; non-specific colocalizations occur at ROIs lacking ^{Cy3B}DNA signal.

(E) Histograms showing dwell time distributions for Cy5-labeled wild-type Lig4 colocalization events, as in Fig. 3. DNA was labeled with Cy3B either at both DNA termini (as in Fig. 3a) or at an internal position near the biotin-streptavidin anchor.

(F) WT Lig4 colocalization FRET and dwell time histograms, as in Fig. 3c-e, in extracts treated with DMSO vehicle or 50 μM NU7441 DNA-PKcs inhibitor. Red lines show dwell time distribution fits generated by maximum likelihood estimation in MATLAB using two-term exponential models. Fit parameters: τ, exponential time constant; amp., amplitude. Figures in brackets represent 95% confidence intervals. Data from three independent experiments.

(G) WT Lig4 dwell times reproduced from Fig. 3d, top panel. Red line shows dwell time distribution fit generated by maximum likelihood estimation in MATLAB using two-term exponential models. Dashed line shows poor fit to a one-term exponential model.

(H) Histograms showing dwell time distributions for WT Lig4 at different 641 nm (Cy5 excitation) powers. Upper panel replicates data from top panel of Fig. 3c; lower panel shows histogram at higher 532 nm laser power. Red lines show dwell time

distribution fits generated by maximum likelihood estimation in MATLAB using two-term exponential models. Fit parameters: τ, exponential time constant; amp., amplitude. Figures in brackets represent 95% confidence intervals. Data from three independent experiments.

(I) Histograms showing dwell time distributions for Cy5-labeled wild-type Lig4 colocalization events (as in Fig. 3) with DNA ends terminated in either 5′ phosphates or 5′ hydroxyls. Extracts were treated with 50 μM NU-7441 to ensure 5′ hydroxyl ends remained unphosphorylated[8].

(J) WT Lig4 colocalization dwell time partitioned by $FRET_E$ (low, <0.1; high, >0.1). ecdf, empirical cumulative distribution function. Data from three independent experiments.

Supplementary Data Figure 3

a

mDBD1

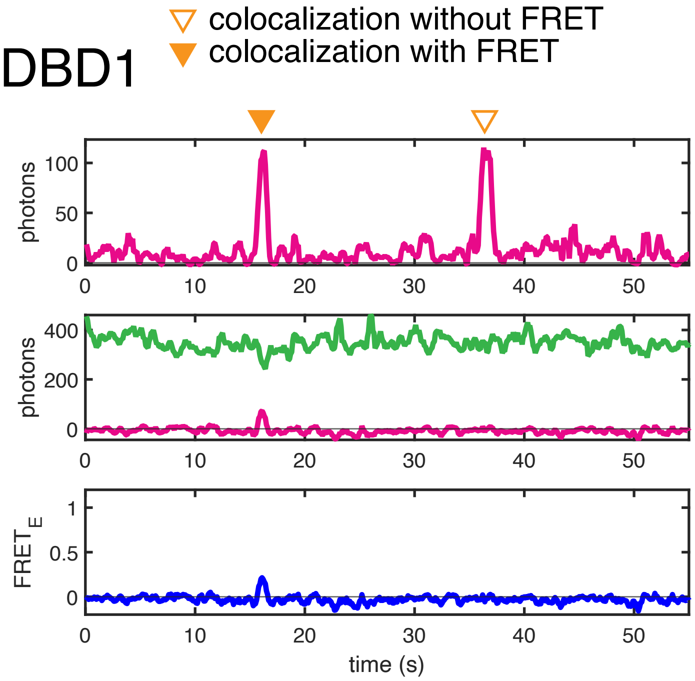

b

mDBD1+2

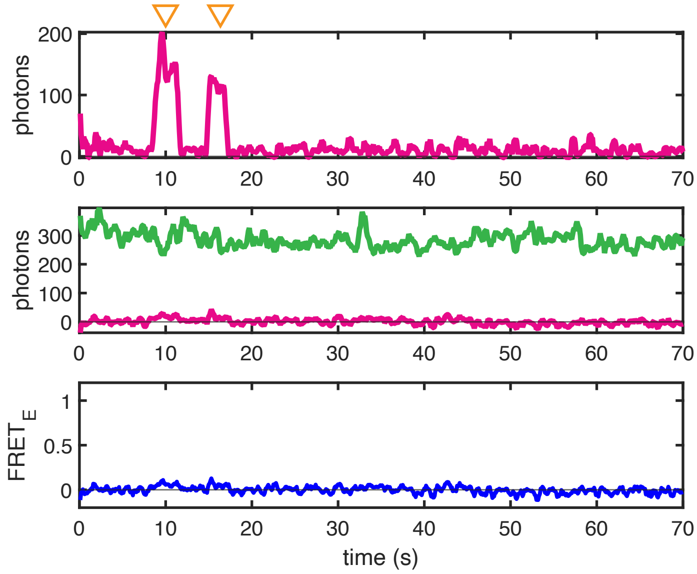

Supplementary Data Figure 3: Example trajectories for Lig4 DNA binding mutants. As in Fig. 3b, representative single-molecule trajectories showing Lig4 colocalization and end binding for mDBD1 (A) and mDBD1+2 (B).

Supplementary Data Figure 4

a

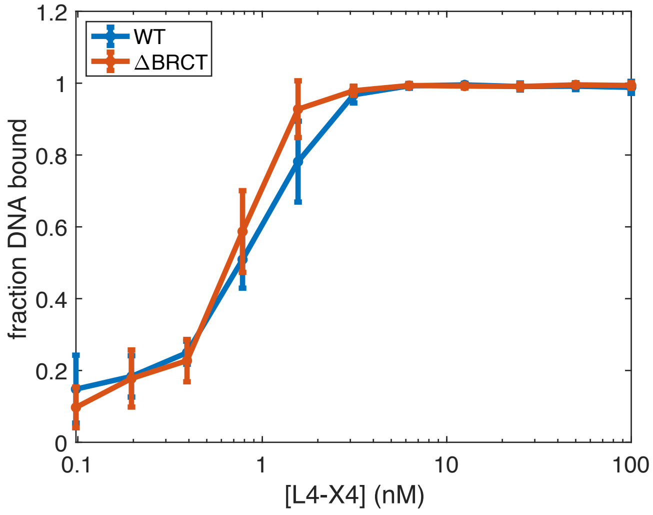

b

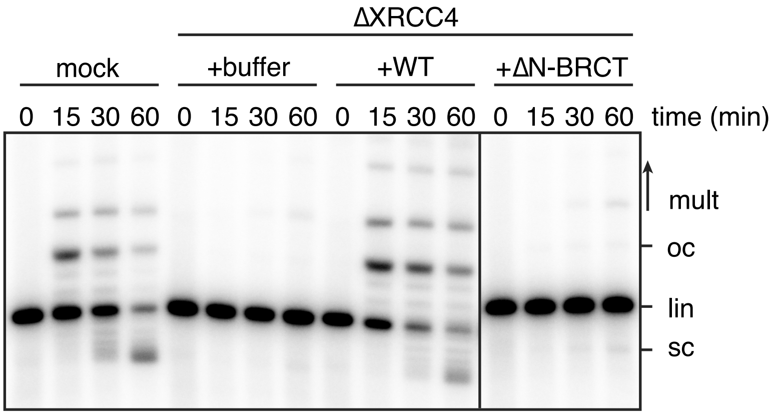

c

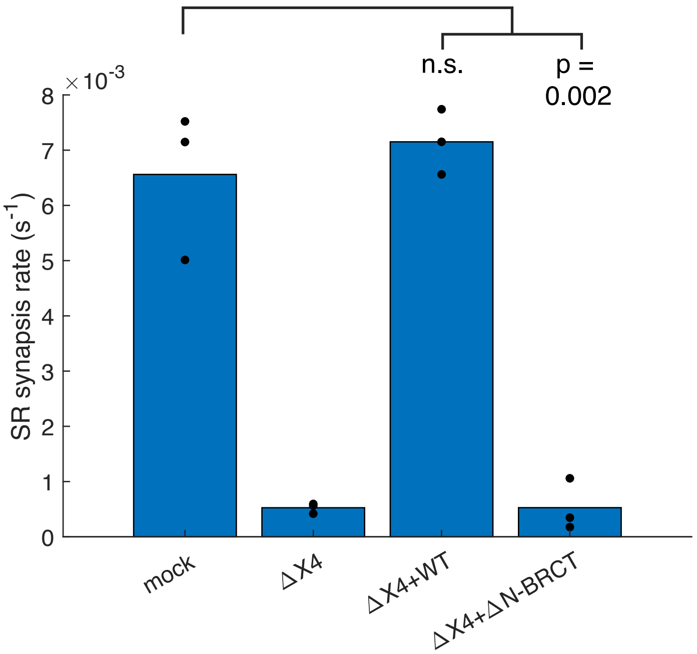

Supplementary Data Figure 4: Characterization of Lig4 ΔN-BRCT variant.

(A) Filter binding assay for DNA binding by WT and ΔN-BRCT Lig4, as in Fig. 1b. WT data are reproduced from Fig. 1b.

(B) End joining assay for WT and ΔN-BRCT Lig4, as in Fig. 1c. Mock, ΔX4, and ΔX4+WT data are reproduced from Fig. 1c.

(C) SR synapsis assay for WT and ΔN-BRCT Lig4, as in Fig. 2c. Mock, ΔX4, and ΔX4+WT data are reproduced from Fig. 2c.

Supplementary Data Figure 5

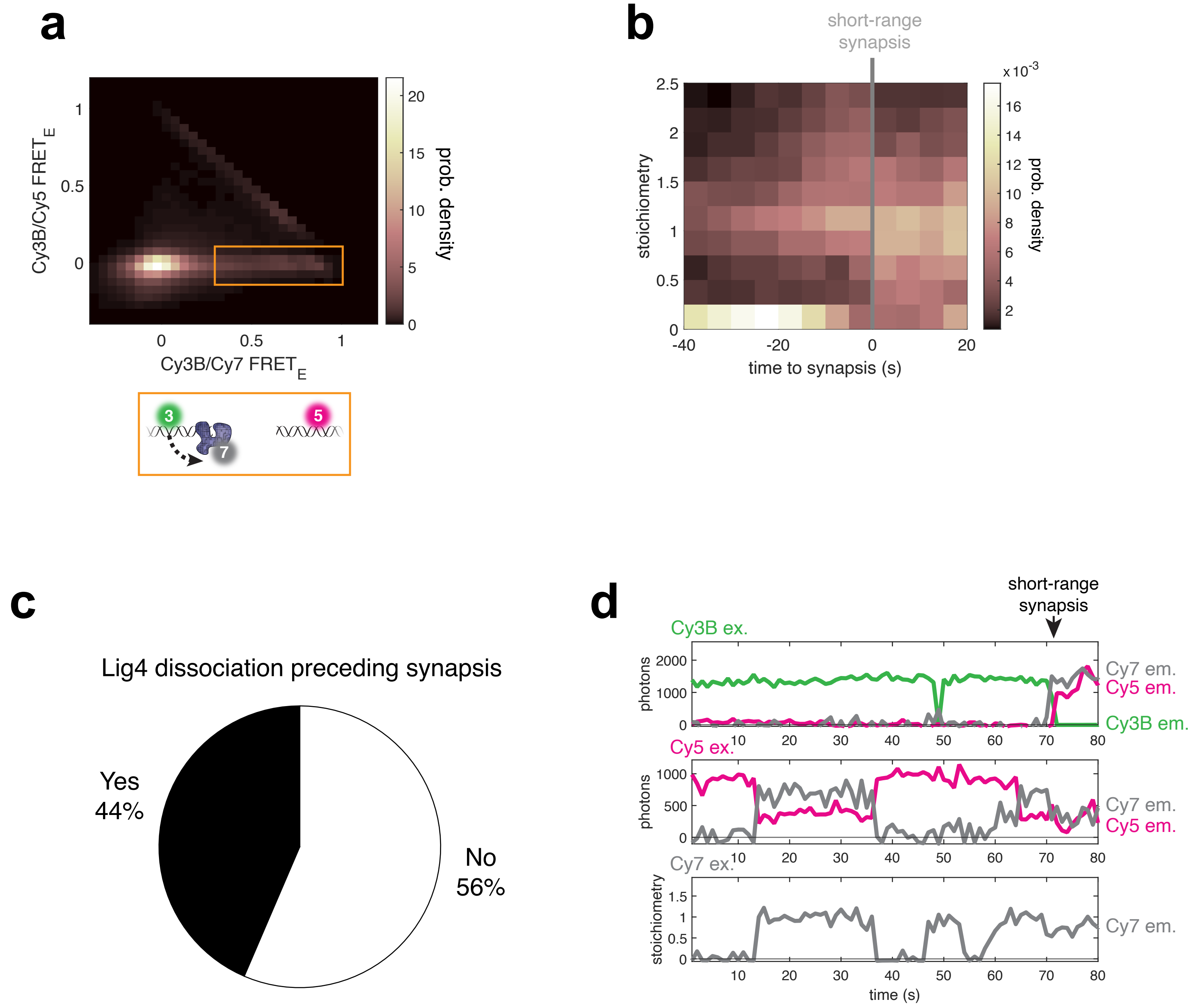

Supplementary Data Figure 5: A single Lig4 binds both DNA ends at the moment of SR synapsis, continued.

(A) As in Fig. 4c, heatmap showing Cy3/Cy5 FRET as a function of Cy5/Cy7 FRET for the experiment shown in Fig. 4. Orange box shows Lig4 DNA binding prior to SR synapsis. Heatmaps contain data from all experimental frames in which neither Cy3B nor Cy5 had photobleached.

(B) Heatmap showing stoichiometry distributions relative to onset of SR synapsis, analogous to Fig. 5b. Gray bar highlights time of SR synapsis.

(C) Pie chart depicting the proportion of SR synapsis events for which a Lig4 dissociation event was detected within a 10 s interval prior to synapsis. Dissociation events were defined as stepwise changes in Cy7 intensity (with 730 nm excitation) of greater than 0.7 stoichiometry units (see Methods).

(D) Example trajectory, as in Fig. 4b, showing a synapsis event in which dissociation of a second Lig4 molecule was not detected within the 10 s before SR synapsis.
