# Supplementary material for: Structural role for DNA Ligase IV in promoting the fidelity of non-homologous end joining
Source: Nat Commun. 2024 Feb 10;15:1250. doi: 10.1038/s41467-024-45553-z (PMC10858965; doi:10.1038/s41467-024-45553-z)
Supplement: Supplementary file 3 — Reporting Summary [file 41467_2024_45553_MOESM3_ESM.pdf]

nature portfolio

Corresponding author(s): Johannes C. Walter
Joseph J. Loparo

Last updated by author(s): 11/01/2023

Reporting Summary

Nature Portfolio wishes to improve the reproducibility of the work that we publish. This form provides structure for consistency and transparency in reporting. For further information on Nature Portfolio policies, see our Editorial Policies and the Editorial Policy Checklist.

Statistics

For all statistical analyses, confirm that the following items are present in the figure legend, table legend, main text, or Methods section.

n/a	Confirmed	
☐	☒	The exact sample size (*n*) for each experimental group/condition, given as a discrete number and unit of measurement
☐	☒	A statement on whether measurements were taken from distinct samples or whether the same sample was measured repeatedly
☐	☒	The statistical test(s) used AND whether they are one- or two-sided<br>*Only common tests should be described solely by name; describe more complex techniques in the Methods section.*
☒	☐	A description of all covariates tested
☒	☐	A description of any assumptions or corrections, such as tests of normality and adjustment for multiple comparisons
☐	☒	A full description of the statistical parameters including central tendency (e.g. means) or other basic estimates (e.g. regression coefficient) AND variation (e.g. standard deviation) or associated estimates of uncertainty (e.g. confidence intervals)
☐	☒	For null hypothesis testing, the test statistic (e.g. *F*, *t*, *r*) with confidence intervals, effect sizes, degrees of freedom and *P* value noted<br>*Give P values as exact values whenever suitable.*
☒	☐	For Bayesian analysis, information on the choice of priors and Markov chain Monte Carlo settings
☒	☐	For hierarchical and complex designs, identification of the appropriate level for tests and full reporting of outcomes
☒	☐	Estimates of effect sizes (e.g. Cohen's *d*, Pearson's *r*), indicating how they were calculated

Our web collection on statistics for biologists contains articles on many of the points above.

Software and code

Policy information about availability of computer code

Data collection	Fluorescence images were acquired using a microscope controlled by Hamamatsu HCImage live version 4.4.0.1 and Labview version 15.0f2.
Data analysis	Single-molecule analysis was performed using an published pipeline (Smith et al., Nat Comm (2019)). Post-processing for visualization was performed using custom Matlab code, available from the authors upon reasonable request.

For manuscripts utilizing custom algorithms or software that are central to the research but not yet described in published literature, software must be made available to editors and reviewers. We strongly encourage code deposition in a community repository (e.g. GitHub). See the Nature Portfolio guidelines for submitting code & software for further information.

Data

Policy information about availability of data

All manuscripts must include a data availability statement. This statement should provide the following information, where applicable:

- Accession codes, unique identifiers, or web links for publicly available datasets
- A description of any restrictions on data availability
- For clinical datasets or third party data, please ensure that the statement adheres to our policy

Source data are provided with this paper. Raw, uncropped gel images are available on Zenodo (doi.org/10.5281/zenodo.10055272). Additional data supporting the findings of this study are available from the authors upon reasonable request.

Research involving human participants, their data, or biological material

Policy information about studies with human participants or human data. See also policy information about sex, gender (identity/presentation), and sexual orientation and race, ethnicity and racism.

Reporting on sex and gender	n/a
Reporting on race, ethnicity, or other socially relevant groupings	n/a
Population characteristics	n/a
Recruitment	n/a
Ethics oversight	n/a

Note that full information on the approval of the study protocol must also be provided in the manuscript.

Field-specific reporting

Please select the one below that is the best fit for your research. If you are not sure, read the appropriate sections before making your selection.

☒ Life sciences ☐ Behavioural & social sciences ☐ Ecological, evolutionary & environmental sciences

For a reference copy of the document with all sections, see nature.com/documents/nr-reporting-summary-flat.pdf

Life sciences study design

All studies must disclose on these points even when the disclosure is negative.

Sample size	Sample sizes were not predetermined. Single-molecule measurements were repeated to allow observation of tens to hundreds of events (exact numbers in figure legends), consistent with typical practice in the field.
Data exclusions	Detection of single-molecule spots was performed automatically using predefined criteria, including intensity, circularity, and distance to neighboring spots.
Replication	All experiments were successfully replicated in at least three independent experiments.
Randomization	Randomization was not included in the design of this study.
Blinding	Blinding was not included in the design of this study.

Reporting for specific materials, systems and methods

We require information from authors about some types of materials, experimental systems and methods used in many studies. Here, indicate whether each material, system or method listed is relevant to your study. If you are not sure if a list item applies to your research, read the appropriate section before selecting a response.

Materials & experimental systems

n/a	Involved in the study	
☐	☒	Antibodies
☒	☐	Eukaryotic cell lines
☒	☐	Palaeontology and archaeology
☐	☒	Animals and other organisms
☒	☐	Clinical data
☒	☐	Dual use research of concern
☒	☐	Plants

Methods

n/a	Involved in the study	
☒	☐	ChIP-seq
☒	☐	Flow cytometry
☒	☐	MRI-based neuroimaging

Antibodies

Antibodies used	Anti-XRCC4 antibody from Graham et al, Mol Cell (2016)

Validation	Rabbit anti-XRCC4 antibody was previously described (Graham et al, Mol Cell (2016)) and validated by immunodepletion and rescue with recombinant protein.

Animals and other research organisms

Policy information about studies involving animals; ARRIVE guidelines recommended for reporting animal research, and Sex and Gender in Research

Laboratory animals	Adult female Xenopus laevis used for egg extract preparations were obtained from NASCO/Xenopus 1.
Wild animals	No wild animals were used in this study.
Reporting on sex	Because this study uses extracts derived from eggs, only female frogs were used.
Field-collected samples	No field-collected samples were used in this study.
Ethics oversight	The Institutional Animal Care and Use Committee of the Harvard Medical Area Standing Committee on Animals approved relevant protocols.

Note that full information on the approval of the study protocol must also be provided in the manuscript.
